# Characteristics, morbidity and mortality factors associated with Intra-Aortic Balloon Pump in Coronary Artery Bypass Graft Surgery patients

**DOI:** 10.12669/pjms.36.6.2649

**Published:** 2020

**Authors:** Sayed Mumtaz Anwar Shah, Nabil I Awan, Azam Jan, Mujeeb Ur Rehman

**Affiliations:** 1 Dr. Sayed Mumtaz Anwar Shah, FCPS. Assistant Professor, Department of Cardiothoracic Surgery, Rehman Medical Institute, Peshawar, Pakistan; 2 Dr. Nabil I Awan, MBBS. Post-Graduate Resident, Department of Cardiothoracic Surgery, Rehman Medical Institute, Peshawar, Pakistan; 3 Dr. Azam Jan, ABTS Head of Department, Department of Cardiothoracic Surgery, Rehman Medical Institute, Peshawar, Pakistan; 4 Dr. Mujeeb Ur Rehman, MS. Senior Medical Officer, Department of Cardiothoracic Surgery, Rehman Medical Institute, Peshawar, Pakistan

**Keywords:** Ejection Fraction, Cardiac Surgery, Coronary Artery Bypass Grafting, Intra-Aortic Balloon Pump, Mortality, Post-op Stroke, Post-op prolonged ICU stay, Post-op prolonged Ventilation

## Abstract

**Objective:**

The aim of our study is to analyze the characteristics, morbidity and mortality of patients requiring an Intra-Aortic Balloon Pump (IABP) in Coronary Artery Bypass Grafting (CABG).

**Methods:**

An analysis was done on the prospectively collected data of 1216 patients who had CABG in our center between July, 2017 and May, 2019 at our hospital. We categorized patients in to an IABP and non-IABP group on the basis of IABP use. We then compared the pre-operative, per-operative and post-operative characteristics of the two groups. We further stratified the patients according to pre-op ejection fraction (EF).

**Results:**

Out of 1216 patients, 135(11.10%) patients required an IABP. 70(51.9%) patients of IABP group and 699(64.7%) patients of non-IABP group had hypertension (p-value 0.0036). 23.0% had previous myocardial infarction (MI) in the IABP group and 13.8% had prior myocardial infarction (MI) in non-IABP group (p-value 0.0463). Among the patients requiring an IABP, 21(15.5%) of patients had normal EF (>50%) (P-value<0.0001), 72 (53.3%) had EF 35-50%, and 41(30.3%) patients had EF<35% (p-value <0.0001). Mortality of IABP group (19.3%) was greater than non-IABP group (2.4%) (P-value 0.00001).

**Conclusions:**

Use of IABP increased as the EF decreased. Rate of post-operative stroke, prolonged ICU stay, prolonged ventilation, re-opening due to bleeding and mortality was seen to be significantly higher in the IABP group.

## INTRODUCTION

One of the most dreadful complication encountered following CABG is low cardiac output syndrome (LCOS). LCOS is defined as a cardiac index of less than 2L/min/m2 in the absence of hypovolemia. LCOS following CABG is known to increase the risk of mortality, stroke, myocardial infarction, pulmonary complications, renal failure, as well as need for reopening.^1^

Moreover, it has been reported that patients with LCOS are at a greater risk of having prolonged ventilator support, prolong stay in intensive care unit (ICU), and in hospital mortality.^2^ In recent years, mortality associated with open heart surgery has significantly reduced due to advances in surgical techniques and better perioperative management. The first line of management is pharmacological therapy. However, if the trend of deterioration cannot be reversed by means of inotropic support, mechanical assist devices (MADs) are used. Amongst MADs, the most frequently used device is the IABP counter pulsation. In selected patients, IABP has been known to reduce mortality.^3^ IABP is a mechanical device in which the balloon inflates just after the closure of the aortic valve causing an increase in diastolic pressures and deflates just before the opening of the aortic valve thereby decreasing the afterload and improving systolic pressures.

Since last 50 years, the IABP is the frequently used MAD. IABPs are applicable for both right and left ventricular support.^4^ The important advantages of the IABP are its minimally invasive nature, its ability to synchronize with the heart, prevention from blood handling outside the body and its easily applicability.^5^

The most common complications associated with IABP are local hematoma formation, local and systemic infection, limb ischemia, balloon rupture/damage, passage failure, aortic perforation/dissection.^6^

## METHODS

This analysis has been approved by The Ethical Review Committee of our hospital with Ref. No: RMI/RMI-REC/Article Approval/25, April 08, 2020 and an informed written consent was taken from all the patients before collecting their clinical data. We prospectively collected the pre-operative, per-operative and post-operative data of all patients in our cardiac surgery database center. From this database, we extracted the clinical data of all the patients that underwent isolated coronary artery bypass grafting between July, 2017 and May, 2019. Patients with concomitant procedures like Atrial Septal Defect (ASD)/Ventricular Septal Defect (VSD) closure or Valvular repair/replacement were excluded.

We divided the total patients into two groups: IABP group and non-IABP group. We then further divided the clinical data of these groups into their pre-operative, per-operative and post-operative characteristics. Analysis was then done between the two groups.

The prolonged ICU stay was defined as an ICU stay for more than 48h. Prolonged Ventilation was defined as a ventilator requirement exceeding 24hours. Stroke was defined as a neurological deficit lasting for more than 72hours. In-hospital mortality was defined as a mortality during the same hospital admission. Follow-up of patients for purposes of this study was done up till hospital discharge

All the required pre-operative routine blood investigations along with chest X-ray, Echocardiography, Electrocardiography, Angiography, Carotid Doppler and Pulmonary Function Tests were done. A routine on-pump cardiopulmonary bypass was performed for all the patients through a midline sternotomy incision under mild to moderate hypothermia. In our study we used ante grade cold-blooded cardioplegia through the aortic root or the venous graft. A left internal mammary artery was used for all the arterial grafts and a reverse saphenous vein was used for venous grafts.

The indications for inserting an intra-aortic balloon pump were:

1. Cardiogenic Shock.
2. Low cardiac output syndrome. (Diagnosed as systolic blood pressures less than 90mmHg in conjunction with signs of tissue hypo perfusion like cold peripheries and decreased urine output despite, high inotropic support that does not respond to fluid challenge.
3. Visibly deteriorating cardiac function.
4. Cardiac Arrhythmia that is refractory to pharmacological therapy.

None of the patients in this study required an IABP pre-operatively and all the patients had an IABP inserted either per-operatively or post operatively. We exclusively used the femoral artery for IABP implantation. In our routine practice, we insert a femoral sheath after induction of anesthesia in all patients who have left ventricular ejection fraction (EF) less than 40%. We used an IABP machine manufactured by Datascope CS-100. All the data was then entered into SPSS software and then analyzed using the Chi-Squared test. A p-Value of less than 0.05 was considered to be significant.

## RESULTS

A total of n=1216 patients fulfilled our inclusion criteria i.e. underwent isolated coronary artery bypass grafting. One hundred thirty-five (11.1%) patients had an IABP insertion and 1081(88.9%) did not require an IABP insertion. Overall, there were 940 (77.3%) male patients and remaining were female patients. The average age in years was 58.01±9.126. Of all the 63.2% hypertensive patients, there was significantly larger number of hypertensive patients 699(64.7) in the non-IABP group (IABP: 51.9% and non-IABP: 64.7% P-value 0.03606). In contrast, a prior myocardial infarction was more commonly seen in the IABP group as compared to the non-IABP group (IABP= 23%, non-IABP=13.8. p-value 0.00463). Out of the total, 500(41.1%) patients had diabetes, 4(0.3%) had COPD, 186(15.3) patients had raised creatinine and 10(0.8%) patients had a history of prior CVA, as shown in Table-I.

**Table-I:** Pre-operative characteristics of all CABG patients.

Variables	Total	IABP	Non IABP	p- value
Total	1216	135(11.10%)	1081(88.90%)	
Age Mean (Y) (mean ±SD)	58.01 ±9.126	58.20	57.99±9.246	
Male	940(77.3%)	107(79.3%)	833(77.1%)	
Hypertension	769(63.2%)	70(51.9%)	699(64.7%)	0.003606
Diabetes	500(41.1%)	51(37.8%)	449(41.5%)	
COPD	4(0.3%)	0%	4 (0.4%)	
Prior MI	180(14.8%)	31 (23.0%)	149(13.8%)	0.00463
Prior CVA (%)	10(0.8)	1(1.7)	9(0.9)	
Raised Creatinine	186(15.3%)	29(21.5%)	157(14.5%)	

Data are shown as n (%), or mean ± standard deviation; **PRIOR CVA:** prior cerebrovascular accident; **IABP:** Intra-aortic balloon pump; **MI:** myocardial infarction; **COPD:** Chronic Obstructive Pulmonary Disease; **CVA:** Cerebro-Vascular Accident.

The per-operative characteristics of all the CABG patients are given in the Table-II. In the IABP group it was found that 21(15.5%) patients had an ejection fraction of >50%, 72(53.3%) patients had an EF of 35-50% and 41(30.3%) had an EF<35%. This was in contrast to 604(55.8) patients in the non-IABP group having an EF of >50%, 412(38.11) patients having an EF of 35-50% and 64(5.9%) patients having an EF <35%. This difference gave a p-value of <0.00001 as showed in Table-III(a). When this same data was viewed after dividing on basis of EF it was seen that there were 626(51.48%) patients with an EF >50% out of which 21(3.35%) needed an IABP. This was compared to patients who had an EF of 35-50% and those patients who had an EF<35%. It was found that 485(39.88%) patients had an ejection fraction of 35-50% (%), out of which the IABP was inserted in 72 (14.9) patients. In contrast, 105(8.6) patients had an EF <35% (%) out of which 41(39.0%) needed an IABP. This came out to have a p-value of <0.00001, shown in Table-III(b).

**Table-II:** Per-operative characteristics of all CABG patients.

Variables	Total	IABP	Non IABP	p- value
LIMA	1028(84.5%)	104(77.0%)	924(85.5%)	
Left Main Stem	111(9.1%)	10 (7.4%)	101(9.3%)	
Avg No. of venous Conduits (mean±SD)	2.53±0.68	2.50 ±0.72	2.54±0.67	
Perfusion Time (minutes) (mean±SD)	96.08±25.01	97.13 ±23.53	95.95 ±25.19	
Cross Clamp Time (minutes) (mean±SD)	53.24±15.95	52.42 ±14.64	53.34 ±16.10	

Per operative characteristics of the CABG patients. Data are shown as n (%), or mean ± standard deviation **LIMA:** left internal mammary artery; **AVG NO:** average number.

**Table-III(a):** Incidence of IABP

Variables	Total	EF >50%	EF 35-50%	EF <35%	p- value
	1216	626(51.48%)	485(39.88%)	105(8.63%)	
IABP	135(11.10%)	21(3.35%)	72(14.84%)	41(39.0%)	<0.0001
Non-IABP	1081(88.89%)	605(96.64%)	413(85.15%)	64(60.95%)	

Association of ejection fraction to IABP. **IABP:** Intra-aortic balloon pumping

**Table-III(b):** Pre-operative ejection fraction of all the CABG patients.

Variables	Total	IABP	Non IABP	p- value
Normal EF >50%	626(51.5%)	21(15.5%)	604(55.8%)	<0.0001
Moderately Reduced EF 35-50%	485(39.9%)	72 (53.3%)	412(38.11%)	
Severely Reduced EF <35%	105(8.6%)	41(30.37%)	64(5.9%)	

Analysis of IABP and Non IABP groups on the basis of pre-operative ejection fraction. **EF:** ejection fraction; **IABP:** Intra-aortic balloon pumping.

In the IABP group, 9(6.7%) patients had prolonged ventilation while 12(1.1%) patient had prolonged ventilation in the non-IABP group (P-value 0.00001). 18(13.3%) patients required prolonged ICU stay in the IABP group while only 59(5.5%) patients with prolonged ICU stay were in the non-IABP group (p-value 0.000396). Post-operative stroke was seen more commonly in the IABP group (IABP: 3(2.2%), non IABP: 6(0.6%), p-value of 0.033101). Out of the 52(4.3%) cases who died in hospital, 26(19.3%) were in the IABP group and 26(2.4%) were in the non-IABP group. P-value of 0.00001 was found to be significant.

## DISCUSSION

Multiple studies over the past 50 years, have shown the beneficial effect of the IABP as a circulatory assist device and the IABP has been the most frequently used mechanical assisted device.^7^ Advantage of IABP application on time is reported from several literatures resulting in improving the patient’s clinical outcome.^8^

There are no new studies on the IABP use in cardiac surgery in Pakistan. The aim of our study was to determine the independent variables resulting in application of IABP, while at the same time we aimed to determine the post-op complications and the prospects of the patients in term of morbidity and mortality in IABP and non-IABP patients following CABG. The commonest variable in our study was low cardiac output syndrome.

For better prognosis of cardiovascular disorders diseases, it is important to protect cardiac system following cardiac surgery. Therefore, to overcome worse outcome, therapeutic strategies are needed in which IABP is better choice for myocardial protection. Moreover, in IABP insertion, variations have to be considered, including duration, advanced age, and IABP support intervention timing without any complications, several physiological and non-physiological factors like the patient’s cardiac function, the anatomy of the coronary arteries, hemodynamic conditions, the size and position of the IABP, the myocardial condition, and reliability and quality of its indication.

**Table-IV:** Post-operative characteristics.

Variables	Total	IABP	Non IABP	p- value
RE-OP for Bleeding	59(4.9%)	10(7.4%)	49(4.5%)	0.142747
Post-OP Stroke >72h	9 (0.7%)	3(2.2%)	6(0.6%)	0.033101
Prolonged Ventilation >24h	21(1.7%)	9(6.7%)	12(1.1%)	0.00001
Prolonged ICU Stay >48h	77(6.3%)	18(13.3%)	59(5.5%)	0.000396
In Hospital Mortality	52(4.3%)	26(19.3%)	26(2.4%)	0.00001

Post-operative analysis of IABP and non IAB groups in CABG patients. Data are shown as RE-OP: re-opening; IABP: Intra-aortic balloon pumping; ICU: Intensive care unit; post-op stroke: Post-operative stroke.

For patients having severely depressed cardiac function following coronary artery revascularization, the IABP plays a phenomenal role in the recovery of the stunned myocardium.^9^ Application of IABPs is an important factor in order to decrease the left ventricular afterload and increase the Coronary blood flow.^10^ This evidence and its easy-applicability have made the IABP one of the most commonly used mechanical circulatory support device in Cardiac Surgery and its use has been increased in cardiac surgery.^11^

The incidence of IABP varies greatly between institutions. For instance, it has reported that the incidence of IABP in CABG patient is 7%.^12^ Another largest study on IABP in CABG patients reported 17%.^13^ In our study, the rate of IABP was 11.10% which was found to be comparable to the worldwide data. It is also reported that advanced age patients need a longer recovery time postoperatively in contrast to younger patients due to which more mechanical support will be required to advanced age patients, especially in the patients with low cardiac output undergoing CABG. Despite of its experienced complications, IABP is still widely used in recent era.^14^ But we did not do the age comparison. In clinical practice, application of IABP is commonly highlighted in advanced age CABG patients.^15^

No significant difference in the two groups with respect to hypertension has been shown.^16^ Conversely, it is also reported that the IABP group had a higher number of hypertensive patients compared to the non IABP group.^17^ In stark contrast, in our data the IABP use was significantly lower in hypertensive patients than in non-hypertensive individuals. We presume this may be because the left ventricular myocardium is trained to pump against a higher afterload in hypertensive patients and is therefore more resilient to the development of LCOS. However, further studies need to be conducted to clarify this difference.

An increase creatinine level was associated with IABP insertion, which was a marker of poor prognosis.^18^ while some studies showed that there was improved renal status with use of IABP resulting in decrease mortality rate.^19^ in our study, raised creatinine was not significantly associated with IABP application.

We found that patients with prior MI were more prone to requiring an IABP. This is similar to the findings reported that there were a significantly higher number of patients in the IABP group who had a prior MI as compared to the non-IABP group.^16^ This phenomenon has been attributed to the effect of the IABP on improving coronary circulation in and its role in reducing the left ventricular stress and cardiac work load). Complete revascularization in patients with hemodynamic instability who had an IABP has been observed.^20^ we also found the improved hemodynamic features after application of IABP. So in our clinical practice, it is worthy to use IABP in hemodynamic instable patient with prior MI patient that will result in better prognosis.

An impaired left Ventricular function has been demonstrated to be an individual risk factor for IABP use. Application of IABPs is an important factor in order to decrease the left ventricular afterload and increase the Coronary blood flow.^21^ The post cardiac surgery use of IABP is beneficial in high-risk patients with low output syndrome.^22^ We found that majority of patients in the IABP had a moderately reduced or severely reduced ejection fraction. However, it was noticed that the IABP was needed in a small number of patients with normal ejection fraction as well. When we analyzed our data with respect to ejection fraction, the trend became clearly visible. The proportion of patients requiring an IABP increased as the ejection fraction decreased. This echoes the findings of the two studies showing the incidence of the IABP usage increased with a falling left ventricular ejection fraction. IABPs are applicable for both right and left ventricular support.^23^ Despite of all the improvements, LCOS still remains one of the major causes of perioperative mortality in CABG.^24^

Although a number of researches have established a connection between patients with left main stenosis and the IABP but we found that there was no significant increased usage of the IABP in patients with left mains stenosis.

As is expected, the IABP was found to be associated with considerably higher morbidity in terms of prolonged Intensive Care Unit (ICU) stay, prolong ventilation time and stroke (lasting >72h) as these patients are in a more critical state than the non IABP group. This finding was also depicted by another study which also reported significantly prolonged ICU stays and ventilation times along with an increased incidence of stroke in patients with an IABP.^16^ Mortality in the IABP group is an interesting detail to consider. In recent years, mortality associated with open heart surgery has significantly reduced due to advances in surgical techniques and better perioperative management.^25^

A higher mortality rate in the IABP group is no surprise and is predicted based on a number of reviews by credible authors. However, it is pertinent to note that in cardiac surgery, often the IABP is used in patients who are deteriorating and in already a very critical state requiring urgent and immediate circulatory support without which survival of these patients would be questionable. Therefore, it follows that instead of observing the mortality rate of IABP, it would be more apt to scrutinize this data as the number of patients the IABP has saved.

IABP application is strongly recommended for the reduction of in hospital mortality rate of patients having cardiac problems.^26^ Significantly different in hospital mortality between the IABP and non IABP group has been reported such as (35%) in IABP group.^15^ In our study, the mortality rate in IABP group was (19.3%) and non IABP group was (2.4%); p-value 0.00001, so it shows a significant difference.

The clinical significances of our study are that instituting IABP per-operatively or postoperatively yielded better outcome in CABG patients because of earlier tackling of end organs perfusion before irreversible damage resulting in decrease morbidity and mortality especially in severely reduced EF and moderately reduced EF and in prior MI patients. We have also reported Post-OP Stroke >72h, Prolonged Ventilation >24h, Prolonged ICU Stay >48h and In Hospital Mortality in IABP group.

### Limitation of study

our study is small sample size. Multi-center studies need to be conducted to determine the hypo-perfusion’s indicator and resolution following CABG surgery. We did not do the male and female comparison. Our study is having uniform determination of outcomes with other studies.

## CONCLUSION

In conclusion, the IABP is more commonly used in patients with reduced ejection fractions and history of prior MI albeit with an increased morbidity and mortality post-operatively.

### Authors’ Contribution

**AJ:** Conceived, designed, statistical analysis & editing of manuscript.

**NIA and MUR:** Data collection and manuscript writing.

**MUR:** Responsible and accountable for the accuracy or integrity of the work.

**SMAS:** Review and final approval of manuscript.
